# Odour enhances the sense of presence in a virtual reality environment

**DOI:** 10.1371/journal.pone.0265039

**Published:** 2022-03-30

**Authors:** Nicholas S. Archer, Andrew Bluff, Andrew Eddy, Chreshall K. Nikhil, Nick Hazell, Damian Frank, Andrew Johnston

**Affiliations:** 1 Agriculture and Food, The Commonwealth Scientific and Industrial Research Organisation, Sydney, Australia; 2 UTS Animal Logic Academy, Faculty of Engineering & IT, University of Technology Sydney, Sydney, Australia; 3 Independent Researcher, Sydney, Australia; 4 Faculty of Engineering & IT, University of Technology Sydney, Sydney, Australia; Universite de Lyon, FRANCE

## Abstract

Virtual reality (VR) headsets provide immersive audio-visual experiences for users, but usually neglect to provide olfactory cues that can provide additional information about our environment in the real world. This paper examines whether the introduction of smells into the VR environment enhances users’ experience, including their sense of presence through collection of both psychological and physiological measures. Using precise odour administration with an olfactometer, study participants were exposed to smells while they were immersed in the popular PlayStation VR game “Resident Evil 7”. A within-subject study design was undertaken where participants (n = 22) walked-through the same VR environment twice, with or without the introduction of associated congruent odour stimuli. Directly after each gameplay, participants completed a questionnaire to determine their sense of presence from the overall gameplay and their sense of immersion in each of the virtual scenes. Additionally, physiological measurements (heart rate, body temperature and skin electrodermal activity) were collected from participants (n = 11) for each gameplay. The results showed the addition of odours significantly increased participants’ sense of spatial presence in the VR environment compared to VR with no odour. Participants also rated the realism of VR experience with odour higher compared to no odour, however odour addition did not result in change in emotional state of participants (arousal, pleasure, dominance). Further, the participants’ physiological responses were impacted by the addition of odour. Odour mediated physiological changes were dependent on whether the VR environment was novel, as the effect of odour on physiological response was lost when participants experienced the aroma on the second gameplay. Overall, the results indicate the addition of odours to a VR environment had a significant effect on both the psychological and physiological experience showing the addition of smell enhanced the VR environment. The incorporation of odours to VR environments presents an opportunity to create a more immersive experience to increase a person’s presence within a VR environment. In addition to gaming, the results have broader applications for virtual training environments and virtual reality exposure therapy.

## Introduction

Smell has been a vital part of human evolution and plays an essential role in our day-to-day lives. The ability to sense odours in the environment affects our day to day decisions, enabling us to judge the edibility of particular items of food, avoid environmental hazards and communicate with others [1]. A smell or odour can induce strong emotional feelings, alter behaviour and can act as a stimulus to the retrieval of autobiographical memory [2–5]. However, in the hierarchy of human senses, smell or olfaction is often underappreciated and inappropriately considered inferior when compared with olfactory performance of other mammals and to the other human sense modalities [6].

Nonetheless, the importance of odour in our everyday lives is recognized by the food, cosmetics and cleaning product sectors, for example, who invest considerable time and resources in the creation of fragrances that best suit their product—and affect the customers’ perception of its desirability [7, 8].

Virtual reality (VR) aims to create an immersive experience which transports the user to another world with established terminology describing various VR aspects. The term *Presence* in VR means how much a user believes that he/she is inside a virtual world. It is generally defined as a user’s subjective sensation of “being there” in a scene depicted by a medium [9]. The term *Immersion* is used to describe an objective measure on how good the hardware and system technology is (e.g. video resolution, audio sampling rates, etc), and how many senses are engaged (e.g. vision, hearing) [10]. The term *Reality* or *Realism* is used to determine how closely the virtual world replicates a real-world counterpart. So usually *immersive* hardware and *realistic* media would be thought to increase the levels of *presence* in a virtual experience, although the link is not always so simple.

As technology has evolved, the level of immersion in VR environments has increased, with huge improvements in visual and aural fidelity, and in the accuracy of head and gesture tracking. The development of head tracking with six degrees of freedom means that users are able to look at any part of the virtual world; spatial audio techniques enable the generation of realistic, immersive sounds that can be linked to simulated objects as they move around a VR scene; and, tactile gamepad controllers and other haptic devices provide mechanisms for simulating touch. All in all, there is a clear trend towards the creation of completely immersive and multi-sensory experiences that enhance a consumer presence in a VR environment. However, despite the recognition of the importance of smell in many aspects of our daily lives, the use of smells in VR remains a novelty and relatively under explored in comparison to sound and vision.

Several publications have assessed the use of smell in a VR environment. Nakamoto & Yoshikawa [11] delivered scents designed to match scenes in a short animated film, and observed that audiences found scenes that featured transitions between contrasting smells were most ‘impressive’. Jones et al. [12], exploring the potential of odour-enhanced virtual environments for military training applications, examined the experiences of 30 participants who played a combat computer game while exposed to three different scents—two of which were congruent with the game scenes (ocean smells on the beach, and a ‘musty’ smell in a fort). They found that in this context, the additional odours did not measurably enhance participants’ sense of immersion.

Munyan et al. [13] assessed the combination of olfactory stimuli on presence in anxiety ridden environments to help with exposure therapy where users performed a simple VR task revolving around losing their keys in a fairground. Results indicated that while participants felt an increase in presence with the smells, there was no measurable increase in levels of anxiety. Ischer et al. [14] developed a 3D immersive environment with advanced control of odour delivery, however did not assess the impact of odour on the VR experience.

Baus and Bouchard [15], explored the effects of pleasant and unpleasant smells on participants’ sense of presence in a virtual environment. They exposed VR participants to pleasant (apple/cinnamon), unpleasant (urine) and ambient scents. While the scents were not directly related to the virtual environment, users that received smells reported an increase in their sense of presence. Oddly, those exposed to the urine (negative) smell described a higher sense of presence than those exposed to the pleasant smell. They speculate that this may have been because the urine smell was perceived as being stronger than the pleasant smell.

Findings from a subsequent study [16] indicated that when the scents matched the scene (e.g. when the ‘pleasant’ scent of cinnamon apple pie was presented in a virtual reality scene featuring two pies on a bench) the ability of participants to detect the odour increased. However, in the later study, the overall sense of presence, as measured by the Independent Television Commission Sense of Presence Inventory [ITC-SOPI] questionnaire [17], did not increase significantly.

Taken together, these studies suggest that odours are more readily perceived when they are matched to visually concordant scents, and that ‘unpleasant’ odours may have a greater effect on the sense of presence. Further, the previous studies generally have limitations with one or more of the following: (i) methodological limitations in study design or development of smells, (ii) crude or simple VR environments (i.e. poor reality) and, (iii) unsophisticated mechanisms to deliver odours to a participant (without the ability to switch rapidly between odours) and therefore, not replicating the reality of how smells are encountered/perceived in the real world.

The aim of the current paper is to examine whether the introduction of smells into the VR environment enhances users’ sense of presence. The current study assessed the impact of odour addition on a participants experience in a VR environment by collecting responses using (i) questionnaires on presence, realism and emotion and (ii) to determine if changes in physiological measures were evident (heart rate, body temperature and electrodermal activity (EDA)). While both of these methods have been previously used in VR research, use of physiological measures to assess increased presence has yielded mixed results to date [18].

Given previous research, we propose that unpleasant odours, presented with concordant visuals in VR should increase the sense of presence. We deliberately chose a VR experience that featured scenes with strong-smelling artefacts (rotten food, etc) to maximise the likelihood of increasing users’ sense of presence. The main outcome measure and hypothesis was the odour delivered in a VR environment would increase a participant’s sense of presence. A secondary measure was that due to the fear and scare elements of the VR experience, we would observe a heightened physiological response when the odour was delivered compared to the same VR environment without odour.

Finally, the methods employed in the current research overcome the limitations of prior research through the use of both a well-developed VR environment and sophisticated delivery of odours to better reflect how smells are encountered in the real world. Understanding the effect of odours on a person in a VR environment will help designers decide whether and when to integrate olfactory cues for different applications (e.g. virtual games, treatment of post-traumatic stress disorder, training, etc).

## Materials and methods

### Overview

In order to explore the impact of adding simulated odours to the VR user experience, we conducted a study in which participants played a commercial VR game enhanced with the controlled addition of synthesized odours. The simulated odours used were developed to be congruent with the VR environment experienced by the participant. A PlayStation VR headset playing the game *Resident Evil 7* was used to create a controlled multisensory experience. The game was augmented with smells generated by an olfactometer, which delivered odour volatiles via a soft plastic tube fixed underneath a participant’s nose to enable free rotation of the head. The olfactometer ensured real-time, precise, computer-controlled odour was delivered to the participant. Until now, only a handful of attempts have been made to integrate odours into the VR field using an olfactometer [19].

### Participants

The research conducted was approved by both CSIRO human ethics committee (2019_031_LR) and University of Technology, Sydney, Human Research Ethics Committee (2013000135 2019–3) and written informed consent was obtained from each participant prior to completing the experiment.

Participants were recruited locally through email adverts (University and CSIRO) and social media posts. Inclusion criteria were for participants between the age of 18 to 60 years, free from current upper respiratory tract infection and free of known fragrance, smell allergies or sensitivity reactions. Additionally, it was recommended that individuals who were sensitive to unpleasant images to not participate in the study. Participants were provided with a gift card to cover time and travel costs associated with completing the experiment.

Participants attended one session where they completed the same section of the Resident Evil VR game twice, once with and without simulated odours. Participants were randomly assigned whether they received the odour in the first or second gameplay. After the first VR gameplay, participants were given a 15-minute break and after each of VR gameplay participants completed a questionnaire about their experience.

### VR and experimental set-up

**Fig 1:**
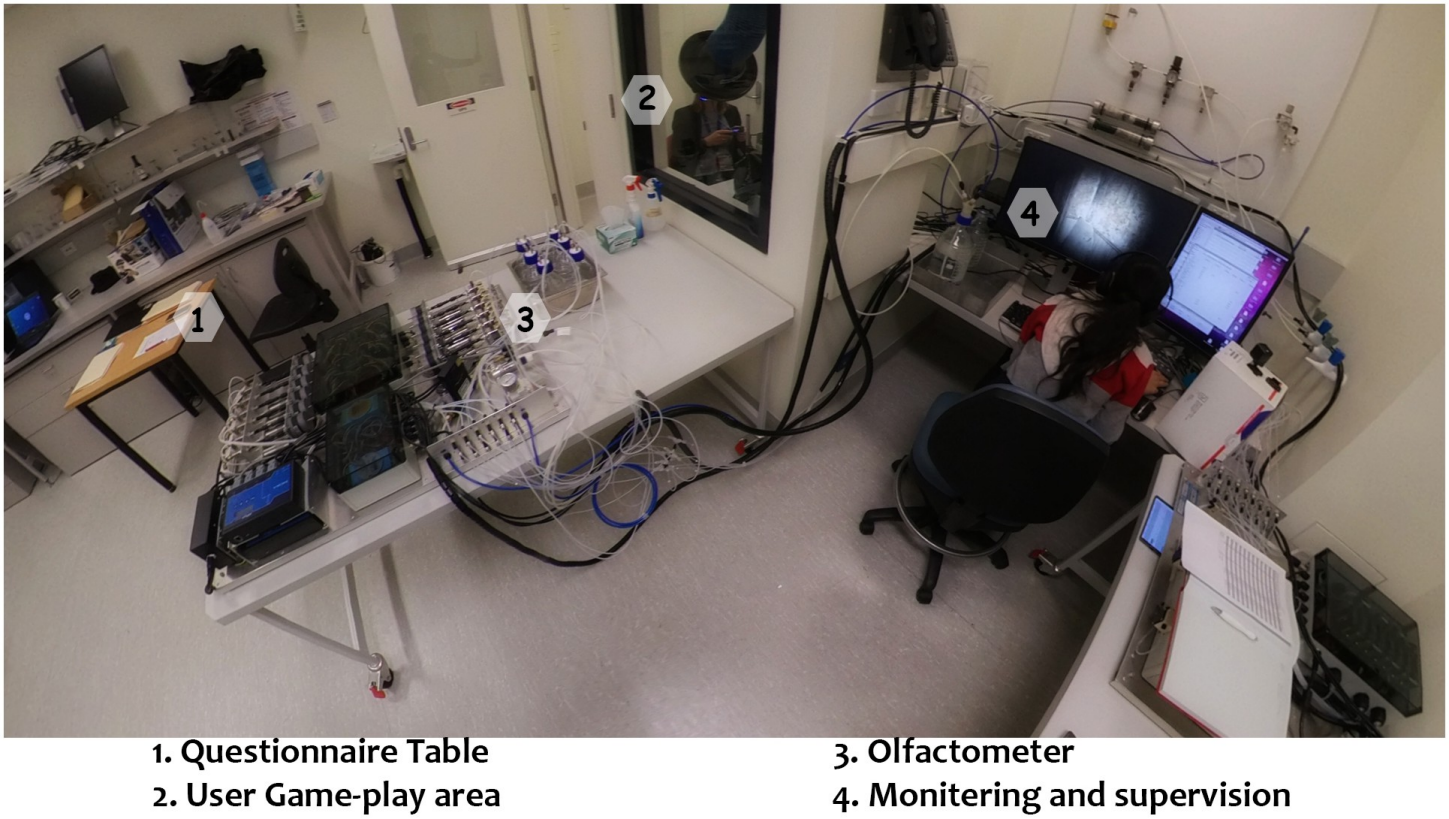
Overview of experimental set-up for the study. Zone 1: participant briefing and post-gameplay questionnaires, Zone 2: the gameplay area, Zone 3: olfactometer, and Zone 4: the monitoring/supervision area.

The experimental setup is shown in Fig 1 and split into different zones. Zone 1 was used to brief participants and complete post-gameplay questionnaires. The user game play area (Zone 2) was in a dedicated small room where a participant sat on a chair and contained:

- The olfactometer mixing and delivery head, connection tubing to a participant’s nose and an extraction fan to remove excess odour.
- A standard PlayStation 4 console, VR headset and wireless controller that were used to run the VR environment and control the game’s character.
- Playstation camera used to capture the PlayStation VR headset motion.
- A subset of participants (n = 11) wore an Empatica E4 Wristband to collect physiological data on heart rate, electrodermal activity (EDA), and body temperature.

Zone 3 contained the odour solutions and olfactometer that controlled delivery of the smells to the mixing and delivery head in the user gameplay area. The monitoring and supervision area (Zone 4) contained:

- A monitor connected to the PlayStation console which simultaneously displayed a 2D version of the VR environment the participant was experiencing.
- A mixing board with a microphone that the experimentalist could talk to the participant through earphones, allowing directions to be communicated to help navigate through the VR environment.
- A monitor connected to the olfactometer with a custom-built interface (software program) for the experimentalist to control the timing of delivery of the odour mixtures.
- A GoPro camera was positioned to record the monitoring and supervision area (2D monitor of VR environment and olfactometer controls) to enable the viewing of each VR at a later time if required (i.e. resolve discrepancies in data).

### Gameplay

Prior to playing the game, participants were provided instructions on how to navigate through the game and completed a short tutorial in a VR environment.

The VR environment was provided by the game *Resident Evil 7* (Capcom). Although classified as a survival horror game, the gameplay used in the experiment was based on exploration and did not incorporate the more extreme horror components of the game (e.g. use of weapons, fighting, reanimated monsters). Use of an established and well-designed VR environment also assisted with reduction of nausea, which can be problematic [13]. During the gameplay, participants were instructed to follow a certain path and to explore the VR environment by the experimental supervisor (communicated through the microphone to the headset worn by the participant).

The length of time to complete the gameplay was 6–10 minutes (~6min for experienced VR/PlayStation player, ~10 minutes for beginners). The gameplay consisted of 3 sections which included:

- Forest walk: a person starts the VR gameplay in a forest and follows a path surrounded by plants and trees. During the forest walk, a participant walks through a swamp, encounters a sculpture made of horse legs and blades and passes a smouldering fire on approaching a house.
- Abandoned house: Participant approaches the apparently abandoned house and walks onto a patio/porch and to the front door of the house. The participant enters the house and walks down the end of a corridor to a kitchen where they open a pot and a refrigerator containing rotten food and cockroaches. The participant then proceeds through the kitchen to another corridor and enters a parlor where they move to a fireplace to pull a handle and open a secret door.
- Basement: the participant moves through the secret door where they climb down a ladder and the ladder breaks and falls to the ground. The participant then walks through the basement into chest/neck-high murky water. The participant moves though the water along a corridor and under a beam where they encounter a submerged rotting head. The gameplay then ends as they walk out of the water.

### Olfactometer and simulated odours

An olfactometer was used to mix and deliver smells to participants at predetermined events during the gameplay. The olfactometer is part of a custom built simultaneous gustometer olfactometer (SGO) that has been previously described [20]. The gustometer component of the SGO was not used in this experiment. The olfactometer component of the SGO uses six pairs of computer-controlled, motorised mass-flow controls to vary air flow through six glass saturators, containing the odour mixture solutions, and six corresponding bypass air flows. Each of the saturator and bypass air-flow pairs can be varied from 0% to 100% of saturator headspace flow at a constant total flow of 150 mL/min. The six streams are then carried to the delivery manifold, where they are switched by vacuum flow into a carrier flow of humidified air (5L/min at 70% RH, 22°C). The odour mixture and carrier air flow are then delivered to the participant through a nasal cannula, fixed below the participant’s nose.

Four odour mixture solutions were prepared and delivered to the participant throughout the gameplay. The odours used were developed to be congruent with the VR environment experienced by the participant but did not try to re-create the complex mixture of volatiles that would be expected in each of the different environments. The odour mixtures were prepared by dilution of pure food grade compounds in water (or used as a neat solution for smoke odour). Each mixture was loaded into the olfactometer to be delivered in separate streams. Two olfactometer aroma streams remained as air blanks and were not used in this study. Table 1 lists the ingredients used to create each odour mixture in the study.

**Table 1:** Odour solutions prepared for loading into olfactometer.

Volatile	MW (*LogP*)	Amount added to 0.5 L H_2_O (mg)	Conc. (1:100)	Odour character
**Odour 1: Forest**				
Cis-3-hexen-1-ol	100.16 (1.3)	400	4	*Green*, *leafy*
**Odour 2: Smoke**				
Liquid smoke Mesquite (Stubbs)	Neat solution	NA	NA	*Smokey*, *sweet*
**Odour 3: Rotten**				
Dimethyl trisulphide	126.26 (1.3)	80	0.8	*Rotten*, *Sulphur*, *cabbage*
Trimethylamine	59.1 (0.3)	100	1	*Fishy*, *rotten*
Ethyl butanoate	116.16 (1.7)	15	0.15	*Fruity*, *sweet*
**Odour 4: Dank**				
2,3,5-trimethyl-pyrazine	122.1 (1.3)	100	1	*Musty*, *potato*
1-octen-3-ol	128.21 (0.65)	100	1	*Earthy*, *mushroom*

MW = molecular weight, LogP = octanol-water coefficient (a measure of fat solubility, if applicable), Conc. (1:100) = diluted concentration (1:100 dilution) delivered to subjects (ppm).

The four odour mixtures were blended in pre-programmed patterns, by the olfactometer, to be congruent with the event being experienced in the VR environment (Table 2). The delivery of each odour mixture was controlled by the experimentalist using a custom software interface pre-programmed to deliver or stop the correct blend of the four odour mixtures. The experimentalist watched the gameplay on a secondary computer display and selected each odour mixture event, by name, to correspond with the current gameplay event. The olfactometer interface recorded a time stamp at each odour mixture event to allow for physiological data measured using the Empatica E4 Wristband (heart rate (HR), electrodermal activity (EDA) and body temperature (Temp)) to be accurately matched to each event in the VR environment. The gameplay between events was conducted with continuous, humidified air flow without delivery of odour mixture.

**Table 2:** List of the gameplay events and the percentage of each odour mixed together and delivered to the participant^a^.

Event	Odour 1: Forest	Odour 2: Smoke	Odour 3: Rotten	Odour 4: Dank
**Start**				
Start next to car	0	0	0	0
Forest Walk	100	0	0	0
Swamp	80	0	0	20
Horse Sculpture	60	0	40	0
Forest Walk	100	0	0	0
Fire/Smoke	20	100	0	0
Near House	20	20	0	0
Hallway	0	0	0	0
Kitchen	0	0	10	0
Casserole	0	0	100	0
Kitchen	0	0	10	0
Fridge	0	0	10	0
No odour	0	0	0	0
Fireplace	0	20	0	0
Basement	0	0	0	60
Water	0	0	0	100
Rotten head	0	0	100	100
All off	0	0	0	0
**Finish**				

^a^Percent of each odour refers to the percent of airflow contributed to by the saturator (containing the odour headspace) for that individual stream of the olfactometer. Individual streams of the olfactometer are combined prior to delivery of a combined smell across all streams.

### Post-gameplay questionnaires

After each gameplay session (i.e. with and with-out odour), participants filled out a questionnaire, which included:

- The Independent Television Commission Sense of Presence Inventory [ITC-SOPI] questionnaire [17], a validated questionnaire split into two parts (Part A and B having six and 38 questions, respectively) with questions rated on a 5 point scale. Answers to different questions are combined to generate scores on different dimensions of presence including spatial presence, engagement, ecological validity/naturalness and negative effects. An additional question was added to the end of the questionnaire “the smells that I experienced matched the virtual environment” which was scored on the same 5-point scale (this question is referred to as “Match question” in this paper).
- Emotional state questionnaire containing three questions using Self-Assessment Manikin (SAM) scale to measure pleasure (unhappy/happy), arousal (calm/anxious) and dominance (weak/powerful) [21].
- A smell recall questionnaire, where participants were shown flashcards of nine events experienced in the gameplay. For each event, a participant had to name the event and rate on a 5-point scale the realism (scale anchors “Did not match the game” to “Perfectly matched”), pleasantness (scale anchors “Very unpleasant” to “Very pleasant”) and strength of the smell (scale anchors “Did not smell” to “Extremely smelly”).
- Three open response questions intended to gather qualitative feedback on their experience. The prompts were: “What did you remember most?”, “What frightened you?” and “Other comments”.
- A short demographic survey to collect age, gender, current work situation and previous experience playing video games and VR.

### Physiological measures

Physiological measures were collected to enable real time assessment of a person’s response to the VR environment as additional assessment of the impact of odour on the user experience in the VR environment. The Empatica E4 Wristband was used to collect physiological data on heart rate, electrodermal activity (EDA), and body temperature. These physiological measures have been validated with traditional laboratory devices used to measure physiological responses [22, 23]. However, as outlined in the introduction, use of physiological measures to assess increased presence has yielded mixed results to date [18] and their use is exploratory and provides the opportunity to build the evidence/validity for their use in future research. In the current experimental set-up, use of a wireless based wrist device was appropriate to ensure that the equipment did not detract from the VR experience through either restriction of free movement of the head (i.e. due the presence of additional cabling) or by a novel/unusual sensation that may impact on a participants response (e.g. devices fitted around the chest to assess respiration).

The participants were seated during the VR gameplay. The wristband was attached to the participant at the start of each gameplay and continuously recorded data during the two gameplays. The event button was pressed to designate the start and end of each gameplay. The arms, wrist and hands were kept relatively still as they held a Playstation game controller with both hands to navigate through the VR environment. This immobilisation of the arm and the E4 Empatica Wristband removed motion artifacts which could alter the accuracy of the physiological measures. This was confirmed by reviewing the accelerometer data from the Empatica Wristband for each participant which showed no/low movement over periods of gameplay. The physiological measures were reviewed on the Empatica web dashboard and downloaded after each session as .csv files.

As outlined above, each participant progressed through the Resident Evil 7 experience at their own unique pace to allow them a sense of agency within the world and further promote their immersion within the world. This freedom means that each ‘smell’ point would occur at slightly different times for each participant. To allow comparison between participants, the data was aligned and truncated to 5 seconds worth as each participant reached the next smell location/event. Five seconds was determined to be sufficient time to receive the odour and for a participant to react to the smell and event.

To truncate and align the data points, the participant’s heart rate, EDA and temperature were collected from the E4 wristband via a number of comma separated values text (.csv) files. The wristband time was synchronised to the current time and the start time and sample duration for each data point was recorded in the .csv file. The application which controlled the olfactometer was also synchronised to the current time and output a .csv file detailing which location (and smell combination) was being output and at which time. A set of Unity C# scripts were created to collate all of the data for each participant around each event and output two measures to describe the 5 second portions after each event: (i) mean: the mean value over the 5 second period after an event and (ii) delta: the difference between the starting measure and the maximum measure from the 5 second period after an event. The collated data was then output into a separate .csv file for further analysis. The measures ‘mean’ and ‘delta’ were used in the analysis as these were the meaningful variables of interest.

### Data analysis

The main outcome variables were the measures from ITC-SOPI for different dimensions of presence: spatial presence, engagement, ecological validity/naturalness and negative effects. Secondary outcomes were responses to emotional state and smell recall questionnaire and the physiological measures (heart rate, EDA and body temperature). There were 22 participants in total, but physiological data was only collected for 11 of the 22 due to equipment malfunction.

With a within-subject study design of 22 participants, the study was adequately powered to detect medium sized effect changes in the main outcome variables (sensitivity analysis using G*Power indicated that the study was powered to detect an effect size of 0.44 with a power of 0.80 and α of 0.05 [24]). Smaller effects may have been identified with a larger sample size. However, from a practical application, the effect of smell to increase a participant’s presence needs to be of a significant level to warrant further assessment and for the potential development of a commercial odour delivery device integrated into VR hardware. Thus, the study was adequately powered to meet the outcomes of the study.

Statistical analysis was conducted in GenStat V19.1 (VSN International, Hemel Hemstead, U.K.). Multivariate analysis of variance (MANOVA) was used to assess the effect of odour on the ITC-SOPI scores (spatial presence, engagement, ecological validity/naturalness and negative effects), responses to emotional state and smell recall questionnaire and the physiological measures. Dependent variables were the presence of odour or control (i.e. with no odour) and also the order of the gameplay (i.e. whether the participant received the odour or control in the first gameplay) and the interaction between the presence of odour and order. To better understand effect size for MANOVA analysis, partial eta squared (η_p_^2^) and 90% confidence intervals of the effect size were determined in excel and The MBESS R Package [25–27], respectively. These effect size measures are reported in S1–S3 Tables that provide expanded information in parallel to the results tables. Contrast analyses of significant interaction effects were performed in R with Cohen’s d effect size and 95% confidence interval calculated using the R package Effsize [28].

For the physiological measures, gameplay events were further categorized in two groups based on the scare factor of fright effect (included casserole, fridge, basement, water & rotten head) or benign (forest, swamp, horse, smoke, near house, hallway, kitchen & fireplace) and included in MANOVA analysis (between subject factors) together with the effects of odour and order.

Graphs were generated in R using the R package ggplot2 [25, 29]. Basic thematic analysis of the qualitative data was conducted in order to identify consistent themes and surface participant suggestions and preferences [30].

## Results

### Overview of participants

A total of 22 subjects participated in the experiment, exploring the same VR environment of Resident Evil 7 on two occasions (with and with-out odour). The mean age for the group was 28.8 years, with 13 male and nine female subjects. Of the 22 participants, 12 were students and 10 had full time employment. Many of the participants had previous experience with virtual reality (n = 13) and only three participants had no experience playing any video games. Only one participant had previously played Resident Evil 7.

### The addition of odours increased spatial presence of VR environment

**Fig 2:**
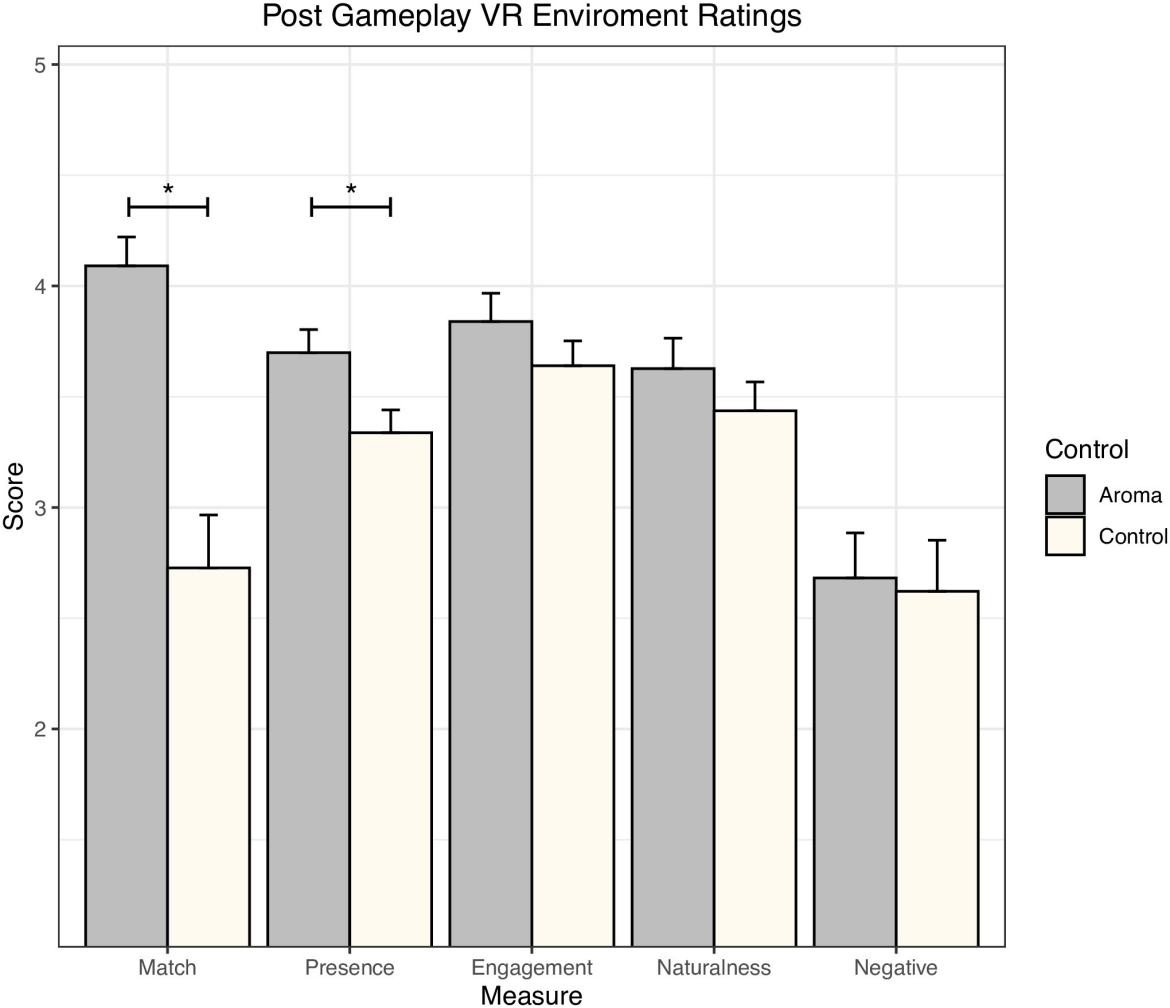
Comparison of ITC-SOPI scores (SEM) post VR gameplay with and without the presence of odour. * *p* < 0.05.

The addition of simulated odours significantly increased participants’ sense of spatial presence in the VR environment (M = 3.7, SD = 0.49) compared to VR with no odours (M = 3.35, SD = 0.51), [F(_1,37_) = 5.72, *p* = 0.022, η_p_^2^ = 0.134, 90% CI [0.011,0.299]] as measured by validated ITC-SOPI (Table 3, S1 Table and Fig 2). Engagement and naturalness scores were higher in the VR gameplay with odour compared to the control with no odour, however the differences were not statistically significant. No difference in negative effects score was observed between the VR environment with or with-out odour. Finally, there was no significant effect of order of the gameplay on measures of engagement, ecological validity/naturalness and negative effects or the interaction between the odour and order.

**Table 3:** Effect of odour addition and order of gameplay on post gameplay questionnaire measures.

Questionnaire	Odour effect				Order effect				Interaction effect*	
	Odour	Control	F-value	P-value	Gameplay 1	Gameplay 2	F-value	P-value	F-value	P-value
**ITC-SOPI**										
Spatial presence	3.70	3.35	5.72	**0.022**	3.41	3.64	2.46	0.125	2.39	0.131
Engagement	3.88	3.66	1.68	0.202	3.75	3.79	0.08	0.778	3.35	0.075
Ecological validity/naturalness	3.61	3.43	0.88	0.355	3.50	3.54	0.04	0.840	2.56	0.118
Negative effects	2.73	2.60	0.21	0.653	2.70	2.63	0.04	0.837	0.42	0.520
**Match question** (5-point scale with anchors: strongly disagree/strongly agree)										
“The smells I experienced matched the virtual environment”	4.10	2.63	32.25	**<0.001**	3.23	3.49	1.00	0.325	1.07	0.308
**Emotional state** (9-point Self-Assessment Manikin (SAM) scale)										
Arousal (Calm/Anxious)	5.25	4.25	3.85	0.057	4.89	4.61	0.29	0.593	0.19	0.663
Pleasure (Unhappy/Happy)	6.29	6.25	0.01	0.938	6.29	6.25	0.01	0.938	0.00	0.981
Dominance (Weak/Powerful)	5.55	5.60	0.01	0.920	5.82	5.32	0.98	0.328	0.69	0.413
**Smell recall questionnaire**										
Pleasantness (5-point scale with anchors: Very unpleasant/Very pleasant)										
Forest	3.39	3.29	0.11	0.741	3.20	3.47	0.80	0.376	2.77	0.104
Horse sculpture	2.57	2.66	0.06	0.805	2.52	2.71	0.24	0.626	0.02	0.899
Outside fire/smoke	3.19	3.02	0.35	0.558	2.96	3.25	1.05	0.313	0.17	0.684
Hallway	2.82	2.80	0.00	0.948	2.75	2.86	0.15	0.698	1.08	0.304
Pot with rotten food	1.56	1.89	1.19	0.283	1.65	1.80	0.24	0.628	1.50	0.229
Fridge	2.00	1.94	0.02	0.884	1.95	1.99	0.01	0.909	0.19	0.665
Fireplace	2.90	2.81	0.17	0.681	2.71	2.99	1.72	0.198	0.30	0.590
Basement water	2.12	2.26	0.18	0.672	1.94	2.44	2.50	0.122	1.21	0.278
Rotten head	1.46	2.04	2.75	0.106	1.69	1.81	0.12	0.736	1.42	0.241
Realism (5-point scale with anchors: Did not match the game/Perfectly matched)										
Forest	3.90	3.42	3.15	0.084	3.44	3.88	2.72	0.107	0.03	0.860
Horse sculpture	3.76	3.27	2.53	0.120	3.40	3.63	0.58	0.450	0.15	0.705
Outside fire/smoke	4.00	3.47	3.31	0.077	3.59	3.88	1.02	0.318	0.12	0.727
Hallway	3.63	3.62	0.00	0.959	3.68	3.57	0.12	0.728	1.99	0.166
Pot with rotten food	4.15	3.66	2.33	0.135	3.87	3.93	0.04	0.846	0.29	0.593
Fridge	4.05	3.77	0.79	0.380	3.82	4.00	0.32	0.574	0.79	0.380
Fireplace	3.80	3.49	1.21	0.279	3.57	3.72	0.29	0.593	0.81	0.373
Basement water	4.14	3.96	0.27	0.607	3.91	4.19	0.73	0.399	0.00	0.976
Rotten head	4.04	3.90	0.16	0.689	3.90	4.04	0.14	0.712	0.18	0.670
Strength (5-point scale with anchors: Did not smell/Extremely smelly)										
Forest	2.55	1.48	15.35	**<0.001**	1.59	2.43	9.62	**0.004**	0.83	0.369
Horse sculpture	3.37	1.47	42.71	**<0.001**	2.24	2.61	1.61	0.212	0.01	0.934
Outside fire/smoke	3.51	1.62	30.17	**<0.001**	2.38	2.75	1.18	0.284	0.02	0.881
Hallway	2.27	1.16	21.86	**<0.001**	1.55	1.88	2.01	0.165	0.75	0.391
Pot with rotten food	4.00	1.82	35.79	**<0.001**	2.87	2.95	0.06	0.814	0.58	0.453
Fridge	3.56	1.73	33.75	**<0.001**	2.88	2.41	2.20	0.146	10.34	**0.003**
Fireplace	3.09	1.35	36.55	**<0.001**	2.13	2.30	0.36	0.555	0.49	0.488
Basement water	3.41	1.50	43.11	**<0.001**	2.27	2.63	1.51	0.226	2.48	0.123
Rotten head	3.51	1.63	29.16	**<0.001**	2.33	2.81	1.86	0.181	0.02	0.889

Table shows mean data from 22 participants,

*Interaction effect assessed Odour*Order interaction. Bold values highlight significant findings.

The additional question “The smells I experienced matched the virtual environment” was rated significantly higher with the odour (M = 4.10, SD = 1.01) compared to without the odour (M = 2.63, SD = 1.23), [F(_1,37_) = 32.25, *p* = 0.001, η_p_^2^ = 0.466, 90% CI [0.258,0.598]] (Fig 2 (Matched), Table 3 and S1 Table) and was independent of the order of gameplay.

No significant differences in the emotional state of the participants were observed (arousal, pleasure and dominance), however there was a trend observed for increased anxiety in the presence of the odour (Table 3 and S1 Table, *p* = 0.057).

The smell recall questionnaire which presented flashcards of events experienced throughout the VR gameplay, found no significant differences when odour was delivered compared to control for each of the nine individual events for pleasantness and realism ratings (Table 3 and S1 Table). However, participants rated the strength of the smells significantly higher (but not too high) for all individual events when the odour was presented in the gameplay compared to the gameplay with no odour—indicating participants did not have difficulty perceiving the smells (Table 3 and S1 Table). Additionally, there was a significant difference between gameplays (order effect) in ratings of the forest strength and an interaction between the presence of odour and order of gameplay on the strength ratings at the fridge event. In the second instance, the strength rating was higher on first gameplay to the fridge event with odour compared to the second (mean scores 4.3 and 2.8, respectively, t(_20_) = 2.4, *p* = 0.028, Cohen’s *d* = 1.01 and 95% CI [0.06–1.95]).

Outcome measures from the smell recall questionnaire were further assessed by ANOVA to determine the effect of the presence of odours or the events combined (rather than comparing each individual event) on ratings of pleasantness, realism and strength (Table 4, S2 Table contains full analysis with each event). No difference in pleasantness ratings were observed with the addition of odour, however, ratings were significantly different between the events. Odour addition had a significant effect on realism ratings (F(_1,374_) = 8.22, *p* = 0.004, η_p_^2^ = 0.022, 90% CI [0.004,0.052], Table 4 and S2 Table), however the event (*p* = 0.115) or interaction between odour and event (*p* = 0.795) did not significantly impact realism ratings. The strength ratings were significantly different between both the presence of odour (*p* < 0.001) and between the different events (*p* < 0.001).

**Table 4:** Effect of odour addition and the events combined on smell recall questionnaire measures.

Factor	Odour effect				Combined event effect		Interaction*	
	Odour	Control	F-value	P-value	F-value	P-value	F-value	P-value
Pleasantness	2.46	2.53	0.46	0.496	14.04	**<0.001**	0.78	0.620
Realism	3.88	3.58	8.22	**0.004**	1.63	0.115	0.58	0.795
Strength	3.21	1.52	250.18	**<0.001**	4.60	**<0.001**	1.54	0.141

Bold values highlight significant findings.

*Interaction effect assessed Odour*Combined event interaction.

### Odour addition altered participants’ physiological response

Physiological measures were collected from participants as they completed the gameplay and measures for each VR event extracted (see materials and methods for details). In addition to odour and order, an additional dependent variable was included in the analysis based on whether an event was benign (e.g. walking through forest) or scary (e.g. a rotten head appearing in submerged water). Due to the smaller number of participants with physiological measures (n = 11, balanced for order), analysis was completed across all gameplay events (rather than individual events) to identify trends from the addition of the odours (Table 5 and S3 Table). The outcome measures were the average measure and the delta change (maximum measure minus start measure) for each heart rate, temperature and EDA.

**Table 5:** Effect of odour addition, order of gameplay and fright on physiological response.

Measure	Odour Effect				Order Effect				Fright Effect				Interaction effect*							
	No Odour	Odour	F-value	P-value	Gameplay 1	Gameplay 2	F-value	P-value	Benign	Scary	F-value	P-value	Odour*Order		Odour*Fright		Order*Fright		Odour*Order*Fright	
													F-value	P-value	F-value	P-value	F-value	P-value	F-value	P-value
**Average HR**	82.06	82.13	0.00	0.956	85.02	79.16	20.16	**<0.001**	81.99	82.25	0.04	0.845	37.38	**<0.001**	0.98	0.323	0.12	0.727	1.27	0.261
**Delta HR**	0.140	0.224	3.68	0.056	0.232	0.131	5.08	**0.025**	0.181	0.183	0.00	0.959	3.52	0.062	0.89	0.347	0.20	0.652	0.04	0.843
**Average Temp**	31.58	32.18	0.01	0.908	31.58	32.18	10.43	**0.001**	31.78	32.04	1.84	0.177	119.30	**<0.001**	0.27	0.601	0.33	0.567	1.78	0.184
**Delta Temp**	0.019	0.015	2.93	0.088	0.019	0.015	2.59	0.109	0.018	0.016	0.48	0.488	1.74	0.188	6.15	**0.014**	6.66	**0.010**	2.46	0.118
**Average EDA**	3.93	2.22	19.39	**<0.001**	3.68	2.47	9.30	**0.003**	2.66	3.73	7.28	**0.008**	27.11	**<0.001**	4.30	**0.039**	2.05	0.154	0.92	0.339
**Delta EDA**	0.095	0.073	1.18	0.278	0.091	0.077	0.46	0.496	0.053	0.135	14.86	**<0.001**	0.00	0.944	0.61	0.436	1.56	0.213	0.28	0.595

HR—heart rate, Temp—body temperature EDA—electrodermal activity. Delta refers to the maximum [measure] minus the starting [measure] for an individual event with measure being one of HR, temperature or EDA. Bold values highlight significant findings.

Overall, the addition of odour to the VR environment resulted in a change in participants physiological response, further confirming odour addition had an impact on the user’s experience. Specifically, the addition of odour resulted in significantly lower (*p* < 0.001) average EDA, compared to no odour. Delta HR was higher and approached significance (*p* = 0.056) in the presence of odour. However, there were clear order effects and interactions between the order of gameplay and odour for most physiological measures (Table 5 and S3 Table: Average HR, Delta HR, Average Temperature and Average EDA). Average EDA was lower in the presence of odour for the first gameplay (1.7 vs 5.23, t(_120_) = 2.9, *p* < 0.004, Cohen’s *d* = -0.51 and 95% CI [-0.15, -0.87]) but no difference was measured in the second gameplay. The Average HR (90.4 vs 81.2, t(_113_) = 3.6, *p* < 0.001, Cohen’s *d* = 0.63 and 95% CI [0.27, 1.00]) and Delta HR (0.334 vs 0.147, t(_66_) = 2.3, *p* < 0.02, Cohen’s *d* = 0.47 and 95% CI [0.11, 0.83]) were higher when the odour was present in the first gameplay compared to no odour. This effect was reversed when odour was present in the second gameplay for Average-HR but was not significant (76.6 vs 83.3). Further, average heart rate was significantly lower when the odour was experienced second (90.43 vs 76.59, t(_77_) = 5.0, *p* < 0.001, Cohen’s *d* = -0.98 and 95% CI [-1.35, -0.60]) further highlighting the impact of order on the physiological effects. Average Temperature was higher in the odour condition compared to no odour (32.8 vs 30.8, t(_108_) = 7.3, *p* < 0.001, Cohen’s *d* = 1.18 and 95% CI [0.78, 1.57]) in the first gameplay. However, this was reversed in the second gameplay with Average Temperature lower when odour was present compared to none (31.3 vs 33.46, t(_100_) = 10.3, *p* < 0.001, Cohen’s *d* = -1.66 and 95% CI [-2.14, -0.83]). Finally, events classified as scary (fright effect) resulted in significantly higher EDA measures for both average EDA (*p* = 0.008) and delta EDA (*p* < 0.001) compared to benign events (Table 5, S3 Table).

### Validity of smells presented and experimental set-up

It was important to verify that the experimental set-up was suitable to assess the effect of odour on the VR environment. Specifically, it is important to understand if the olfactometer was delivering smells that a participant was able to perceive, and further, if the odours perceived were congruent with the objects and experiences in the VR environment. This information can be gained from post-hoc analysis of the data and can provide assurance for the validity of the experimental set-up.

The fact that participants rated the strength of the smells (Table 3, Smell recall questionnaire) significantly higher when odour was presented compared to when they were not presented indicates that the odour mixtures delivered by the olfactometer were detected by participants at each event.

There are three different pieces of evidence that the odour mixtures prepared were congruent with the VR environment. Firstly, the question “the smells that I experienced matched the virtual environment” was rated significantly higher with the odour compared to without the delivery of odour (*p* < 0.001) (Fig 2 (Matched), Table 3) and was independent of the order of gameplay (*p* = 0.325) suggesting the smells were perceived as congruent to the VR environment by the participants. Secondly, Realism ratings (from the smell recall questionnaire) were not significantly higher when odour was delivered compared to control for each of the nine individual events (Table 3). The realism ratings were further assessed by ANOVA to determine the effect of the odour or the gameplay events combined on realism rating. A significant effect of odour was observed compared to control (Table 4, *p* = 0.004), however the event (*p* = 0.115) or interaction between odour and event (*p* = 0.795) did not significantly impact realism rating. This shows that overall, the presence of the odours did have an impact on how realistic participants found the VR environment, independent of any one event. Finally, pleasantness ratings (from the smell recall questionnaire) were separated into two groups based on whether the event was a negative or neutral smell/experience (Negative = Horse sculpture, Pot with rotten food, Fridge, Basement water, Rotten head; Neutral = Forest, Outside fire, Hallway, Fireplace). All negative events had lower mean pleasantness scores compared to neutral events (mean values 1.9 and 3.1, respectively) suggesting congruent smells were delivered in the experiment.

### Qualitative feedback

Qualitative feedback gathered from participants via the three written, short-answer questions (“What did you remember most?”, “What frightened you?” and “Other comments”), were analysed in order to identify factors that appeared to enhance or detract from the overall experience and the sense of immersion. In general, participants indicated that the use of smells was interesting and provided an experience which was quite unique, eg. “[This was] a very interesting experience, have not experienced anything like it before”.

In terms of suggested improvements, there were comments that the level of smells should be able to be adjusted. Because of the somewhat extreme nature of the *Resident Evil* environment, many smells were quite strong, and three comments suggest that providing some kind of calibration for individual preferences should be considered, eg. “I find it very intense, way too much odour for my liking”.

Several participants also suggested that providing a wider range of smells, including more ‘pleasant’ smells beyond the horror genre, would make the experience more attractive for them personally (eg. “There [were] no good smells so the engagement…could be better with both good and bad smells”). Along similar lines, one participant expressed the view that the experience with aromas was “more immersive but probably less pleasant”.

Finally, one participant suggested the soft plastic tube that delivered the aromas under the nose should be “designed to wear more comfortably”.

## Discussion

The findings of this study show that users’ sense of presence is enhanced when simulated smells are introduced into a VR environment. Presence is a multi-dimensional construct and the ITC-SOPI measures four different determinants of presence including Spatial Presence, Engagement, Ecological Validity (Naturalness) and Negative Effects (Nausea) [17]. Spatial Presence received a significant increase from the addition of odour, indicating that participants felt more immersed in the virtual environment with the addition of odour. Further, the effect size observed from the addition of aroma on spatial presence was large (n_p_^2^ = 0.134) [27] and therefore of a significant contribution to a participants VR experience to support the development of commercial odour delivery devices integrated into VR hardware. There was a mild (non-significant) increase in both engagement and naturalness when odour was present which may indicate that the smells were well suited to the experience (ie they didn’t detract) but that the horror narrative (engagement) and audio/visual depiction (naturalness) of the game environment were more important factors than the smells themselves. Finally, the presence of odour did not alter the participants’ experience of negative effects (motion sickness), which is a surprisingly positive result taking into account some of the disgusting experiences with smells that were presented (e.g. maggots in casserole dish and rotten head). The ITC-SOPI results demonstrate that the addition of odour increased the participants’ feeling of presence in the virtual environment, without any additional negative side effects. Furthermore, the overall significant increase in realism ratings further confirms the effect of odour addition to enhance the VR experience.

The addition of odour to the VR experience resulted in differences in the participants physiological responses for HR, body temperature and EDA, compared to no odour. Considering the order effect, EDA was lower, while HR and body temperature were both higher in the presence of odour. The changes in the physiological measures observed are most likely due to the odours enhancing the fear and scare elements of the VR experience compared to no odour (e.g. fight or flight response of the sympathetic nervous system). There may be alternate mechanism(s) causing the physiological effects observed, for example parasympathetic nervous activation where the odours could be perceived as relaxing, decreasing stress, or evoking an arousal response. However, these alternate mechanisms are not consistent with: (i) the context the odours are presented in a VR environment (i.e. horror theme), (ii) the observed increase in heart rate which has been shown to increase with unpleasant odours in preparation for a defensive action [31] and (iii) the order effects that were observed (i.e. if odour was relaxing it should equally effect all gameplays and not just have an effect on first exposure). Further research is required to identify/confirm pathway(s) responsible for the changes in physiology in response to odour addition to the VR environment.

Surprisingly, significant order and order*odour interactions were observed across many of the physiological measures. In most circumstances, the order effect only had an impact on participants’ experiences in the VR environment in the first gameplay and not the second. This suggests that odour addition in some circumstances is only effective on the first exposure to the VR environment (i.e. when the situation/environment is novel to a participant). If a participant has already been exposed to the VR environment and/or can anticipate coming events, the addition of smell may not have an effect on enhancing their experience through physiological changes. While not the main focus of the current study, this order effect requires further attention. A similar experiment utilising participants who are conditioned to a VR environment prior to testing in the experiment would enable further exploration of order events. If the effect of smell addition on users’ experience in VR are not sustained beyond initial exposure there are implications for how odours should be deployed in practice.

Additionally, the current study delivered congruent smells to a participant compared to a control with no smell. Therefore, the current study cannot rule out that the delivery of any smell (i.e. a non-congruent smell) may also result in increasing a participant sense of presence in the VR environment. While other studies suggest that congruency of the smell is important feature to increase a participants sense of presence compared to a non-congruent smell [32], this needs to be further explored using an experimental set-up which would include a non-congruent smell study arm.

The findings presented in this paper show the use of smells in virtual reality experiences can enhance users’ sense of presence and the perceived realism of the virtual environment. We selected a popular and well-known horror game as the focus of our study as this genre seemed well matched to the kinds of primal, physical experiences that the addition of smells could enhance. While the game was a useful test bed for more ‘extreme’ smells, we believe the findings from this study demonstrate that the use of smells in virtual environments should be seriously considered for situations where presence and realism are critical. Beyond gaming, the use of carefully designed odours in virtual training environments for emergency services (fire fighting for example), military personnel, hazardous chemical response teams, etc. is likely to increase users’ sense of immersion and therefore lead to improved outcomes.

The addition of smell to digital devices is clearly intriguing, as there have been many attempts over the years to create commercially viable products and some high-profile failures. A recurring question is one of value. Specifically, what does the addition of smell bring to digital devices, and does this justify the extra complexity and expense? A key theme is the attempt to increase people’s sense of presence and/or the perceived realism of environments present on screen or using virtual reality hardware. The findings of the current study provide additional evidence to support the value of the use of odour in a VR environment.

There are several challenges faced by those who seek to commercialise products that add scents to digital devices. First, the size of devices suitable for use in home or office currently limits the palette of smells that can be reliably delivered. Small devices have been created, but these can dispense only a limited range of scents [33, 34]. Second, the molecular nature of scents means that they appear and disappear very slowly in comparison with the instant response of screen pixels and digital audio. These challenges can be addressed at least to some degree, but until the efficacy of smell to enhance users’ experience is well established, the motivation to invest the necessary time and effort into commercial development is likely to remain low. Given the difficulty in producing high-quality odour delivery devices for widespread use, it would seem that larger-scale training simulations, gallery and theme-park installations are perhaps the most likely to make use of larger scent delivery devices (similar to the device used in the current study and that presented in Ischer et al. [14]) in the short term.

The issue of perceived ‘realism’ of smells is an interesting one for virtual environment designers to consider. We see parallels here with foley—the practice of creating environmental sounds for films. Foley artists are employed to add sounds in post-production—that is, after scenes have been shot. These will include sounds such as footsteps, car doors closing, rain, wind and other environmental sounds. In general, the sounds that are added to the scenes are not those that are recorded by microphones on location. Rather, foley artists source sounds from elsewhere and painstakingly edit, process and time them to fit the video. A key point is that foley sounds are crafted not only for realism but also to enhance the scene and for emotional effect.

When examining and evaluating audiences’ experiences with virtual environments combined with synthesised smells, we therefore suggest that it is likely to be more useful to ask whether the smells enhanced the experience or provided greater emotional impact than whether they were perceived as being accurate or authentic.

For virtual environment designers used to working with animated graphics and sound, we note that the timing of odour delivery and dissipation is likely to be a challenge. Images and sounds can be made to appear and disappear almost instantly, and the ‘teleportation’ of users in a virtual world is technically trivial. For odours, however, there are physical limits to how quickly air can be delivered and removed. This means that transitions from scene to scene need to be choreographed with care to ensure odours transition with the current location. It may mean that certain game mechanics (such as teleportation) will need to be adjusted slightly to allow for the delayed olfactory delivery.

## Conclusion

The results of this study provide further evidence of smells influence upon users’ experience and sense of presence in VR environments. We have explored the use of odours in the somewhat extreme virtual environment of a VR game in the horror genre, with scenes featuring intense smelling objects such as rotten food, smoke and a rotting head. Whether the effects we identify here hold in more realistic or everyday environments will require further assessment. However, we believe that the evidence we have gathered here suggests that smells could effectively enhance the realism of virtual reality training environments, which may often need to simulate ‘extreme’ situations somewhat similar to those explored in this study.

## Supporting information

S1 Table Effect of odour addition and order of gameplay on post gameplay questionnaire measures. Expanded Table 3 from the manuscript containing the standard deviation, effect size (partial eta squared, ηp2) and 90% confidence interval of the effect size for each measure and interaction.
(XLSX)

S2 Table Effect of odour addition and the gameplay events combined on smell recall questionnaire measures. Expanded Table 4 from the manuscript containing the means of the individual events and, the standard deviation, effect size (partial eta squared, ηp2) and 90% confidence interval of the effect size for each measure and interaction.
(XLSX)

S3 Table Effect of odour addition, order of gameplay and fright on physiological response. Expanded Table 5 from the manuscript containing the standard deviation, effect size (partial eta squared, ηp2) and 90% confidence interval of the effect size for each measure and interaction.
(XLSX)
